# Blood transcriptome analysis revealing aging gene expression profiles in red panda

**DOI:** 10.7717/peerj.13743

**Published:** 2022-07-22

**Authors:** Jing Luo, Liang Zhang, Fujun Shen, Li Luo, Lei Chen, Zhenxin Fan, Rong Hou, Bisong Yue, Xiuyue Zhang

**Affiliations:** 1 Key Laboratory of Bio-resources and Eco-environment, Ministry of Education, College of Life Science, Sichuan University, Chengdu, China; 2 Sichuan Key Laboratory for Conservation Biology of Endangered Wildlife, Chengdu Research Base of Giant Panda Breeding, Sichuan, China

**Keywords:** Red panda, Transcriptome, Aging, Immune alternations, Disease prevention

## Abstract

The red panda is an endangered forest species distributed on the edge of the Qinghai Tibet Plateau. The species has been conserved in ex-situ in many countries and its survival is threatened by many diseases. Its immune system is vulnerable to age-associated alterations, which accumulate and result in a progressive deterioration that leads to an increased incidence of diseases. We identified 2,219 differentially expressed genes (DEGs) between geriatric (11–16 years) and adult individuals (4–8 years), and 1690 DEGs between adults and juveniles (1 year). The gene expression and functional annotation results showed that the innate immunity of red pandas increases significantly in geriatric individuals, whereas its change remains unclear when comparing adults and juveniles. We found that the adaptive immunity of red pandas first increased and then decreased with age. We identified CXCR3, BLNK, and CCR4 as the hub genes in the age-related protein–protein interaction network, which showed their central role in age-related immune changes. Many DNA repair genes were down-regulated in geriatric red pandas, suggesting that the DNA repair ability of the blood tissue in geriatric red pandas is significantly reduced. The significantly up-regulated TLR5 in geriatric individuals also suggests the possibility of enhancing the vaccination immune response by incorporating flagellin, which could be used to address decreased vaccine responses caused by age-related declines in immune system function. This work provides an insight into gene expression changes associated with aging and paves the way for effective disease prevention and treatment strategies for red pandas in the future.

## Introduction

In 2015, the IUCN Red List announced that the red panda (*Ailurus fulgens*) would be upgraded from “vulnerable” to “endangered” (Sharma, Belant & Shaner, 2019). Red pandas have attracted much attention within the scientific community because of their decreasing wild population (Zhang et al., 2017). At present, many conservation institutions have begun artificial feeding and breeding research of red pandas, to cultivate artificial populations for the future rejuvenation of the wild red panda population. Red pandas are vulnerable to a variety of mammalian infectious and parasitic diseases that seriously threaten their populations, particularly neonatal and geriatric red pandas (Delaski, Ramsay & Gamble, 2015). Like other mammals, the immune system can resist the daily attacks of foreign organisms and pathogens, and work with other systems to maintain the stability of the internal environment and physiological balance (Pawelec, 1999). However, a basic understanding of the red panda immune system is under-researched and rarely reported.

Aging is an inevitable natural process of every organism. Age-related decline in immune function is known as immunosenescence (Franceschi, Bonafe & Valensin, 2000). Immunosenescence implies a decrease in cell-mediated immune function and humoral immune response (Weiskopf, Weinberger & Grubeck-Loebenstein, 2009). With age-associated alterations, the immune system degenerates naturally and immunity also declines, which means a progressive loss of physiological integrity, impaired function, and an increased incidence of infectious diseases (Aw, Silva & Palmer, 2007; Lopez-Otin et al., 2013). Therefore, a better understanding of the basic mechanisms of immune dysfunction occurring with aging will help delay or even reverse the adverse effects of immunosenescence. Statistics show that the mortality rate of geriatric and adult red pandas is significantly higher than juvenile red pandas, and the main cause of death is cardiovascular disease, followed by kidney disease (Delaski, Ramsay & Gamble, 2015). Most of these chronic diseases are age-related, but the underlying molecular mechanisms that lead to disease susceptibility remain largely unknown (Niccoli & Partridge, 2012). Blood is the main component of the animal immune system and plays a key role in the animal immune system. Meanwhile, blood interacts with every organ and tissue of the body, and the gene expression of blood cells that circulate through various tissues and organs can experience unique changes in response to disease or injury in other tissues and cells of the body (Brodin & Davis, 2017; Liew et al., 2006). In addition, many genes previously thought to be expressed only in non-blood tissues are also expressed in peripheral blood cells (Liew et al., 2006). Peripheral blood is an easily accessible experimental material and provides a large biosensor library in the form of gene transcripts. Peripheral blood can detect the responses to changes in macro and micro environments as gene transcript levels change, thus blood transcriptome analysis can be used to indicate physical health, evaluate drug efficacy, identify disease biomarkers, and reveal the pathogenesis of disease (Mohr & Liew, 2007; Peters et al., 2015; Stephenson et al., 2021). The purpose of this study was to determine which genes and pathways in the red panda blood transcriptome are differentially expressed and the potential genes related to disease development with aging, to pave the way for exploring the mechanism of age-related immune changes.

## Material and Methods

### Sample collection

Peripheral blood samples of twelve red pandas were obtained during routine examinations at the Chengdu Research Base of Giant Panda Breeding, China (age and gender shown in Table S1). All red pandas were sampled in 2018. This study was approved by the Chengdu Institute of Biology Animal Use Ethics Committee (NO. 2018008). After collection, fresh blood samples were immediately stored in the PAXgene Blood RNA Tubes, and then stored at the −80 °C refrigerator until RNA extraction.

### Library preparation and sequencing

Threefold red blood cell lysis buffer (TIANGEN, Beijing, China) was added to lyse erythrocytes and Sorvall Legend X1 (Thermo Scientific, Waltham, MA, USA) was used to centrifugate peripheral blood mononuclear cells (PBMCs). Total RNA was extracted by the M5 Universal RNA Mini Kit tissue/Cell RNA rapid Extraction Kit (Mei5 Biotechnology Co. Ltd, Beijing, China). After assessment and purification of RNA quality, samples with RIN (RNA integrity number) values higher than 7.5 were used for library construction and sequencing. Sequencing libraries were constructed by using NEBNext^®^ Ultra™ RNA Library Prep Kit for Illumina^®^ (NEB, Ipswich, MA, USA). All libraries were sequenced by using the Illumina NovaSeq 2000 platform with a paired-end sequencing length of 150 bp (PE150). Library construction and sequencing were performed by Novogene Bioinformatics Institute (Beijing, China).

### Quality control and sequence alignment

The red panda genome assembly and reference annotation were downloaded from https://www.dnazoo.org/assemblies/Ailurus_fulgens. After sequencing, we obtained 12 sets of transcriptome data. For obtaining high-quality reads, raw data were refined by Trimmomatic (Bolger, Marc & Bjoern, 2014) to remove index, adapter and low-quality sequences. Parameter were set to SLIDINGWINDOW:5:20, LEADING:5, TRAILING:5, MINLEN:50, and others used default parameters. We used FastQC (http://www.bioinformatics.babraham.ac.uk/projects/fastqc/) to generate quality reports for evaluating the quality of the raw and clean reads. High-quality paired-end reads from 12 libraries were mapped on the red panda reference genome using HISAT2 (Kim, Langmead & Salzberg, 2015), separately. We used default parameters for scoring and the matching process. SAM files, generated by Hisat2, were sorted by SAMtools (Li et al., 2009) to generate BAM files.

### Differentially expressed genes analysis

Raw read counts were the input in DESeq2 (Love, Huber & Anders, 2014) for each gene and were extracted by featureCounts (Liao, Smyth & Shi, 2014). *P* values adjusted (padj) ≤ 0.05 & —log2 Fold Change—≥ 1 was used to determine significant DEGs.

### Functional annotation and gene enrich analysis

The gene sequences were searched against NCBI non-redundant (NR) database using BLASTX (Altschul et al., 1997) with a typical cutoff *E*-value of 1E-5. KEGG pathway annotation was performed on KAAS. Gene ontology (GO) was applied with the Blast2GO (Conesa et al., 2005) to obtain annotations of unknown genes. To classify functions of the DEGs, we performed GO enrichment analysis using GOstats (Falcon & Gentleman, 2007) and KEGG pathway enrichment analysis using clusterProfiler (Yu et al., 2012).

### Cluster analysis of Mfuzz transcriptome expression pattern

The R package Mfuzz (Kumar & Futschik, 2007) clusters and visualize genes with similar expression patterns, and explores the dynamic changes of different genes over time. Mfuzz uses a new clustering algorithm of fuzzy C-means algorithm, called soft clustering. Clustering methods are often based on hard clustering, in which a gene is assigned to a cluster. In contrast, soft clustering methods can assign a gene to several clusters, and it is more noise robust and can avoid a priori pre-filtering of genes (Futschik & Carlisle, 2005). We divided all the genes into 15 clusters, the minimum score (membership) threshold was set to 0.25. We then performed GO enrichment analysis for each cluster.

### protein–protein interaction network analysis

protein–protein interaction analysis can check the relationship between proteins expressed by DEGs, and search for hub genes. We first inputted immune-related DEGs into the STRING protein interaction database (Szklarczyk et al., 2019) and then generated protein–protein interaction networks. Next, we downloaded the TSV text file and imported it into Cytoscape (Smoot et al., 2011) software to visualize the annotated protein–protein interaction network.

## Results

### Transcriptome sequencing and sequence alignment

Twelve peripheral blood samples (PBMCs) of red pandas were sequenced by Illumina HiSeq™ 2000 using pair-end sequencing. 311 million raw pair-end reads and 296 million clean reads were obtained after quality control by removing adapter and low-quality reads. HISAT2 results showed that high-quality reads had a good alignment with the reference genome of the red panda, with an average alignment rate of 87.35% (Table S1). Our data have been deposited into the CNGB Sequence Archive (CNSA) (Guo et al., 2020) of the China National GeneBank DataBase (CNGBdb) (Chen et al., 2020a) with an accession number CNP0002175.

### Identification of age-related differentially expressed genes

**Figure 1:**
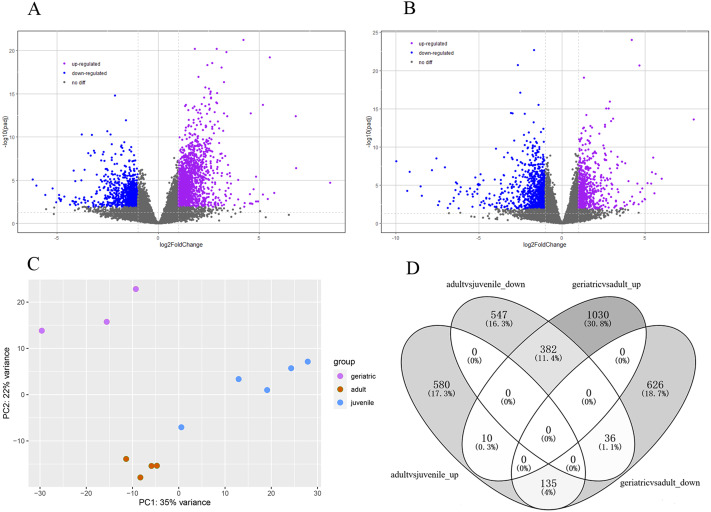
Differentially expressed genes. (A) Volcano plot between geriatric and adults showing the distribution of gene expression plotted against log2 fold change for each gene. Purple and blue dots indicate differentially expressed genes (FDR ≤ 0.05), black dots indicate non-differentially expressed genes. (B) Volcano plot between adults and juveniles. (C) Principal component analysis (PCA) of gene expression between 12 samples. (D) The comparative distribution of 563 common DEGs.

The lifespan of wild red pandas is about 12 years and up to 18 years in captivity (Ali Khan & Kamal Naidu, 1981). The samples were categorized into juvenile, adults and geriatric by age according to a previous study (Delaski, Ramsay & Gamble, 2015). Principal component analysis (PCA) results showed that juvenile, adult, and geriatric individuals could be divided into three groups in different dimensions (Fig. 1C), indicating that there were differences in gene expression patterns between the three age groups. In total, 2219 genes were significantly differentially expressed in red panda blood samples between geriatric and adult individuals. Among the 2219 DEGs, 1422 genes were up-regulated while 797 genes were down-regulated in geriatric compared to adult individuals (Fig. 1A). We found that 1690 genes were significantly differentially expressed between adult and juvenile individuals. Among the 1690 DEGs, 725 genes were up-regulated while 965 genes were down-regulated in adults compared to juveniles (Fig. 1B). We found that 563 genes were differentially expressed in the two comparisons (Fig. 1D). All DEGs are shown in Supplementary 2.

### Gene ontology enrichment analysis of differentially expressed genes

To comprehensively understand the biological role of DEGs, we conducted GO enrichment analysis. Results showed that for the up-regulated genes between geriatric and adult individuals, almost all the significantly enriched GO terms in the biological process category were associated with the innate immune system (Supplementary 1 and Supplementary 2), such as neutrophil activation (GO:0042119), leukocyte activation involved in immune response (GO:0002366), leukocyte degranulation (GO:0043299), and defense response (GO:0006952). Obsolete cytoplasmic vesicle part (GO:0044433) and signaling receptor binding (GO:0005102) were the most significantly enriched GO term in the cellular component and molecular function categories, respectively. For down-regulated genes, the most significantly enriched biological process GO term was chromosome segregation (GO:0007059). Regulation of CD40 signaling pathway (GO:2000348) and B cell activation (GO:0042113) were also enriched. Condensed chromosome (GO:0000793) and molecular adaptor activity (GO:0060090) were the most significantly enriched GO term in the cellular component and molecular function categories, respectively (Supplementary 1 and Supplementary 2).

For the up-regulated genes between adults and juveniles, DNA replication (GO:0006260), chromosomal region (GO:0005654) and ATP-dependent microtubule motor activity (GO:0008574) were the most significantly enriched GO term in the biological process, cellular component and molecular function categories, respectively (Supplementary 1 and Supplementary 2). For down-regulated genes, the significantly enriched GO terms in the biological process category were mainly associated with organ development, such as animal organ development (GO:0048513), and multicellular organism development (GO:0007275). Cell periphery (GO:0071944) and sequence-specific DNA binding (GO:0043565) were the most significantly enriched GO term in the cellular component and molecular function categories, respectively (Supplementary 1 and Supplementary 2).

### Pathway enrichment analysis of differentially expressed genes

Hypergeometric test with a *P*-value cutoff of 0.05 was used as the criteria for pathway detection. Down-regulated genes between adults and juveniles were significantly enriched in four pathways, including Parathyroid hormone synthesis, secretion and action (ko04928), Axon guidance (ko04360) and Cholinergic synapse (ko04725). Down-regulated genes between geriatric and adult individuals were significantly enriched in six pathways, including Intestinal immune network for IgA production (ko04672) and B cell receptor signaling pathway (ko04662); more details can be found in Supplementary 1.

### Immune related genes

**Figure 2:**
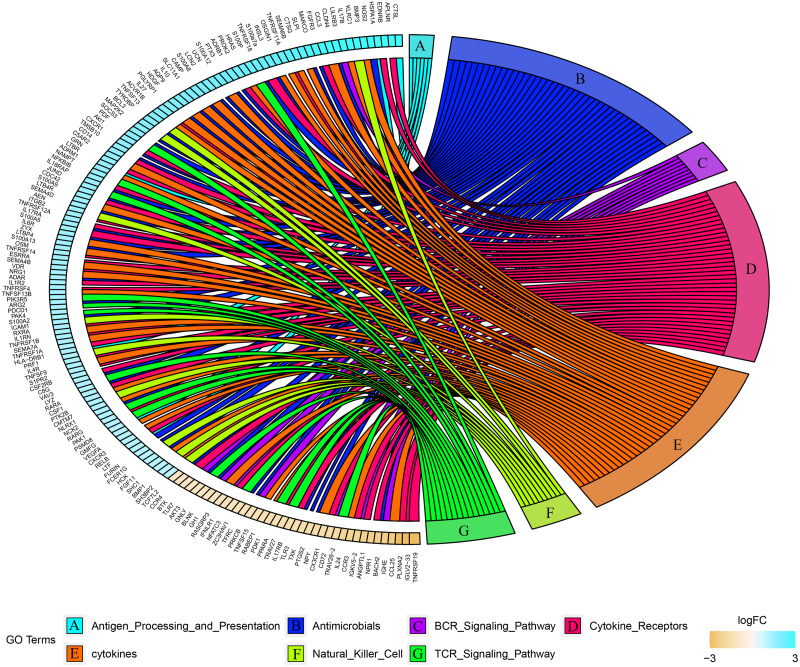
Chord diagram of categories of immune DEGs between geriatric and adults.

**Figure 3:**
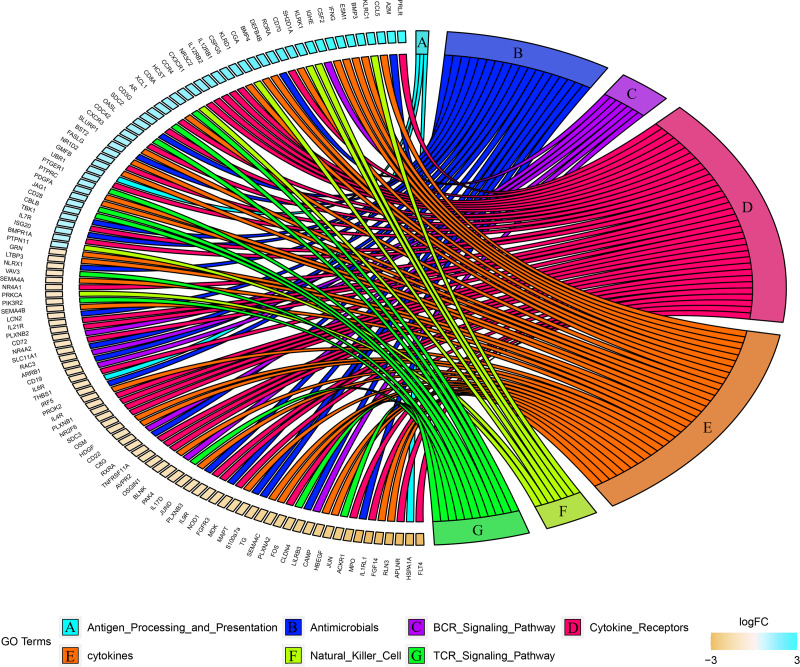
Chord diagram of categories of immune DEGs between adults and juveniles.

Combined with the ImmPort database, we detected 163 immunity related DEGs between geriatrics and adults, and 116 immune related DEGs between adults and juveniles. Genes (PRKCB, BLNK, IGLV2-33, BTK, CD72, IGKV5-2, IGHE, RASGRP3) related to the BCR signaling pathway were down-regulated when comparing geriatrics to adults, and almost all genes belonging to natural killer cell were up-regulated (Fig. 2). Similarly, almost all genes belonging to natural killer cell were up-regulated when comparing adults to juveniles and genes (HSPA1A, THBS1) involved in antigen processing and presentation were down-regulated (Fig. 3). The expression level of CXCR3, BMP3, and KLRC1 continued to increase with increasing age, and the expression level of BLNK, CD72, PLXNA2 continued to decrease with increasing age.

### Cluster analysis of Mfuzz transcriptome expression pattern

**Figure 4:**
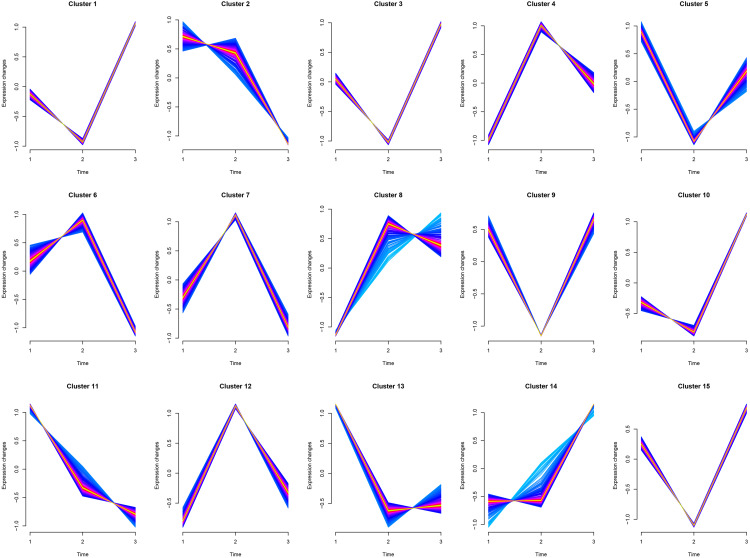
Cluster analysis of Mfuzz transcriptome expression pattern. Trend analysis clusters genes with similar expression patterns according to their temporal profiles (juvenile, adult, geriatric). GO enrichment analysis for each cluster is shown in Table S7.

Cluster1, Cluster3, and Cluster10 were related to leukocyte activation, and all genes experienced a small decline and then a significant rise. Cluster6 was related to humoral immunity, where genes (MEF2C, PRKCB, ATM, TFRC, BANK1, MSH2, PELI1, EXO1, STAP1) experienced a slight rise followed by a significant decline. Cluster8 was related to cellular immunity, where genes (CD8A, CD3G, IL12RB1, XCL1, CBLB, CD6, KLRK1, ZNF683, EOMES, TBX21, CCL5, PRLR, IL7R, PTPN22) experienced a significant rise followed by a slight decline. Cluster5, Cluster11, and Cluster13 were mainly involved in animal organ development, where there was a significant decline from juveniles to adults and little difference between adults and geriatrics. Cluster14 was related to response to external stimulus where there was little difference from juveniles to adults, but a sharp increase between adult and geriatric individuals (Fig. 4). More details of each cluster are shown in Supplementary 2.

### Protein–protein interaction network of differentially expressed genes

We performed a protein–protein interaction analysis of 33 immune-related DEGs that were common in the two comparisons (Table 1). CXCR3, BLNK, and CCR4 were at the key position of the interaction network (Supplementary 1).

**Table 1:** Thirty-three immune-related DEGs that were common in the two comparisons.

Gene symbol	Gene description	Log2FC		Type
		Adult-juvenile	Geriatric-adult	
*HSPA1A*	heat shock protein family A (Hsp70) member 1A	−4.65605793	2.267117457	Antigen Processing and Presentation
*C8G*	complement C8 gamma chain	−1.323993749	1.208984757	Antimicrobials
*CLDN4*	claudin 4	−2.239129897	3.148813288	Antimicrobials
*JUND*	JunD proto-oncogene, AP-1 transcription factor subunit	−1.439104566	1.564296492	Antimicrobials
*LCN2*	lipocalin 2	−1.076628541	2.11372877	Antimicrobials
*NLRX1*	NLR family member X1	−1.035408556	1.164222236	Antimicrobials
*S100a7a*	S100 calcium binding protein A7A	−1.958883689	2.373999596	Antimicrobials
*SLC11A1*	solute carrier family 11 member 1	−1.115382804	1.968902512	Antimicrobials
*BLNK*	B cell linker	−1.406416584	−1.1307455	BCR Signaling Pathway
*CD72*	CD72 molecule	−1.088614858	−1.733981295	BCR Signaling Pathway
*IGHE*	immunoglobulin heavy constant epsilon	2.954148316	−2.377432397	BCR Signaling Pathway
*LILRB3*	leukocyte immunoglobulin like receptor B3	−2.243575969	3.25109424	BCR Signaling Pathway
*APLNR*	apelin receptor	−4.326781357	4.650156681	Cytokine Receptors
*CCR4*	C-C motif chemokine receptor 4	1.644823712	−1.052058544	Cytokine Receptors
*CX3CR1*	C-X3-C motif chemokine receptor 1	1.713708318	−1.672821717	Cytokine Receptors
*CXCR3*	*C* − *X* − *C* motif chemokine receptor 3	1.303070848	1.105685341	Cytokine Receptors
*FGFR3*	fibroblast growth factor receptor 3	−1.728045213	2.931152467	Cytokine Receptors
*IL4R*	interleukin 4 receptor	−1.242601623	1.226363294	Cytokine Receptors
*IL6R*	interleukin 6 receptor	−1.172252092	1.423821312	Cytokine Receptors
*PLXNA2*	plexin A2	−1.903555435	−3.668139961	Cytokine Receptors
*RXRA*	retinoid X receptor alpha	−1.330663396	1.262262761	Cytokine Receptors
*TNFRSF11A*	TNF receptor superfamily member 11a	−1.335786001	2.560094347	Cytokine Receptors
*BMP3*	bone morphogenetic protein 3	3.739604433	3.60074447	cytokines
*CAMP*	cathelicidin antimicrobial peptide	−2.321472105	2.036598684	cytokines
*GRN*	granulin precursor	−1.004731552	1.655891229	cytokines
*HDGF*	heparin binding growth factor	−1.302057881	1.944179971	cytokines
*OSGIN1*	oxidative stress induced growth inhibitor 1	−1.367947542	2.399301935	cytokines
*OSM*	oncostatin M	−1.292596566	1.396710988	cytokines
*PROK2*	prokineticin 2	−1.174311847	2.229834977	cytokines
*SEMA4B*	semaphorin 4B	−1.066977692	1.364958783	cytokines
*KLRC1*	killer cell lectin like receptor C1	3.806902216	3.528593103	Natural Killer Cell
*PAK4*	p21 (RAC1) activated kinase 4	−1.430913333	1.28321432	TCR Signaling Pathway
*VAV3*	vav guanine nucleotide exchange factor 3	−1.035902369	1.208782905	TCR Signaling Pathway

## Discussion

Previous research has indicated that age has a wide-ranging effect on gene expression (Charruau et al., 2016). In this study, the up-regulation of some genes related to organ development and cell proliferation in juvenile individuals, such as FGF14, FOS, HBEGF, and JUN, indicating that the juvenile phase of the red panda is dominated by physical and organ development. Varying stimuli experienced by the organism will cause different types of DNA damage (Chatterjee & Walker, 2017). Accurate and timely DNA repair can offset this damage and restore the stability of the genome. With aging, DNA damage becomes more serious, but the changes in DNA repair capacity appear to be species- and tissue-specific (Chen et al., 2020b). We found many DNA repair genes (INO80, PRIMPOL, RAD51AP1, KLHL15, ERCC6, RNF138, ANKRD32, FANCD2, SMC4, FANCB, PRKDC, SPIDR, SETX, RIF1, SMG1, USP45, DTL) experienced a rise and then a marked decline from the juvenile phase to the geriatric phase (Cluster7). Therefore, our results indicate that the DNA repair ability of blood tissue in geriatric red pandas is significantly reduced. We also observed significant age-related down-regulation in telomere maintenance genes DKC1, and ATM. This may highlight the importance of telomeres in the health and aging of red pandas.

In this study, genes related to neutrophil activation, inflammatory chemokine CCL3 and chemokine receptors CXCR3, and CXCR1 were up-regulated in geriatrics, indicating that geriatric red pandas are in a state of inflammation. A pervasive feature of aging is a constitutive pro-inflammatory environment with persistent low-grade innate immune activation that may augment tissue damage caused by infections in elderly individuals (Franceschi et al., 2018; Sanada et al., 2018; Shaw et al., 2010). Cytokines and chemokines are major culprits in the development of chronic inflammation and the immunosenescence process (Chung et al., 2019). Chemokines and their receptors are important mediators of leukocyte trafficking and leukocyte recruitment during inflammation (Belay et al., 2002). Furthermore, hyperactivity of neutrophils contributes to an inflammatory condition in the tissues (Mortaz et al., 2018). In addition, we found that interleukins (IL10, IL27, IL4R, IL6R, IL1RN, IL1R2) were up-regulated in geriatric individuals. IL10 has potent anti-inflammatory and regulatory activities in most immune processes of infection and disease, limiting the immune response to pathogens and thereby preventing damage to the host (Ip et al., 2017; Saraiva & O’Garra, 2010). Another geriatric up-regulated interleukin, IL-27 is an anti-inflammatory cytokine that suppresses pro-inflammatory Th17 cells and induces anti-inflammatory IL-10 producing T regulatory 1 cells (Frangieh et al., 2020; Rahiman et al., 2013). Furthermore, IL4R, IL6R, IL1RN, and IL1R2 all have anti-inflammatory effects (Ocsko et al., 2018; Opal & De Palo, 2000; Rabe et al., 2008). The up-regulation of the above-mentioned genes may enhance anti-inflammatory cytokine responses and limit injurious sustained, or excessive inflammatory, reactions.

### Innate immune changes

Innate immunity includes neutrophils, macrophages, dendritic cells, natural killer cells, antimicrobial agents, opsonins, and cytokines (Rosenzweig & Holland, 2011). Neutrophils are the most abundant leukocytes, defending against pathogens by degranulation and the release of neutrophil extracellular traps (Pruchniak, Arazna & Demkow, 2013). Neutrophil degranulation pathway genes, such as ITGB2, CD14, ITGAD, CXCR1, LCN2, S100A9, and SLC11A1, were at first down-regulated in juveniles and then up-regulated in geriatrics. TNFR superfamily members (TNFRSF1B, TNFRSF4, TNFRSF9, TNFRSF14) play important roles in both innate and adaptive immunity (Collette et al., 2003; He et al., 2017) and were at first down-regulated in juveniles and then up-regulated as individuals aged. TNFRSF4 contributes to the activation and survival of neutrophils (Jin et al., 2019) and TNFRSF9 is indispensable for innate immunity against *Candida albicans* infection (Tran, Nguyen & Kwon, 2021). TNFRSF14 is involved in innate mucosal defense as its activation leads to activation of neutrophil effector functions, including respiratory bursts, degranulation, and interleukin-8 release (Haselmayer et al., 2006; Shui & Kronenberg, 2013).

Several important genes involved in antiviral response (OASL, ISG20, STAT4) were significantly up-regulated in adults. OASL is an interferon-inducible antiviral protein that can boost innate host defense by enhancing the sensitivity of RIG-I activation (Zhu, Ghosh & Sarkar, 2015). ISG20, as an interferon-inducible 3′–5′exonuclease, inhibits replication of several human and animal RNA viruses and plays an important role in the host’s defenses against pathogens (Degols, Eldin & Mechti, 2007; Zhou et al., 2011). STAT4 promotes activation of RIG-I signaling to initiate more type I IFN production in antiviral innate immunity (Zhao et al., 2016). Natural killer cells have been known to be an essential part of the innate immune system (Averdam et al., 2009). Our results indicated that killer cell lectin-like receptors (KLRC1, KLRD1, KLRK1, KLRG1, KLRF1) were all up-regulated in adults. However, IRF5, MX1, and TLR5, playing crucial roles in innate immune were down-regulated in adults. Interferon regulatory factor 5 (IRF5), a member of the interferon regulatory factor (IRF) gene family, is a key transcription factor for the activation of innate immune responses in the Toll-like receptor signaling pathway (Takaoka et al., 2005). Myxovirus resistance 1 (MX1), an interferon-induced gene that encodes a GTPase, is considered to be an important part of innate immune responses for antiviral host defense (Spitaels et al., 2019). Toll-like receptors (TLRs) are a key component of the innate immune system that recognize microbial infection and trigger antimicrobial host defense responses (Akira & Takeda, 2004). TLR5 binding to bacterial flagellin activates signaling through the transcription factor NF-kappa B and triggers an innate immune response to the invading pathogen (Yoon et al., 2012). Taken together, we consider that the innate immunity of red pandas increases significantly in geriatric individuals, whereas its changes from the juvenile phase to adulthood remain obscure, which are probably influenced by many factors during the rapid physiological development period. Likewise, the immune changes are also complex.

### Adaptive immune changes

The adaptive immune system consists of T cells, B cells and their antigen-specific receptors (TCR and BCR) (Martin, 2014). T cell receptor signaling pathway genes (IL12RB1, XCL1, TBX21, CD8A, IL7R, JAG1, CD3G) experienced an up-regulation and then a down-regulation from the juvenile phase to geriatric phase. The T cell-specific T-box transcription factor (TBX21) is vital to the regulation of the immune system because this factor induces the development and differentiation of T (H)1 as well as contributing to cell-mediated immunity against intracellular pathogens (Chung et al., 2003; Suttner et al., 2009). CD8A that encodes a critical coreceptor of cytotoxic T cells—the CD8 alpha chain of the dimeric CD8 protein—is critical for cell-mediated immune defense and T-cell development (Xu et al., 2014). Signaling mediated by interleukin 7 receptor (IL7R) is essential for normal T-cell development and homeostasis. IL7R inactivation causes severe combined immunodeficiency in humans (Jiang et al., 2005; Puel et al., 1998). Furthermore, genes (THEMIS, PTPRC, BMP4, PRDM1, CD6, CD28, EOMES, PRLR, IFNG) related to T cell activation were up-regulated in adults. Protein tyrosine phosphatase receptor type C (PTPRC), also known as CD45, is an essential regulator of T and B cell antigen receptor-mediated activation (Al Barashdi et al., 2021). CD6 facilitates adhesion between T cells and antigen-presenting cells and modulates thymocyte selection and T lymphocyte activation (Santos, Oliveira & Carmo, 2016). We further found Interferon-regulatory factor 4 (IRF4) was down-regulated in geriatric individuals. IRF4 is induced after T cell receptor (TCR) stimulation, and the level of its expression correlates with the strength of TCR signal (Harberts et al., 2021). The transcription factor IRF4 controls activation and differentiation of CD8+ T cells and is essential for the function of CD8+ memory T cells (Yao et al., 2013). Consequently, our results showed that the cellular immunity of red pandas at first increased in juveniles and then decreased with increasing age

B cell receptor signaling pathway genes (STAP1, PRKCB, MEF2C, BANK1) experienced an up-regulation then down-regulation from juvenile to geriatric individuals. PRKCB plays a central role in propagating NF-Kappa B signaling and cell proliferation downstream of the BCR and is vital to the formation of germinal centers and plasma cells (Tsui et al., 2018). MEF2C has a critical function in mature B cell survival and proliferation after BCR stimulation (Wilker et al., 2008). B cells can differentiate into plasma cells and secrete immunoglobulins against the pathogen (Geng et al., 2020). We found genes (ATAD5, IGJ, IGHE) related to immunoglobulin were up-regulated in adults. ATAD5 deficiency has been shown to reduce B cell division and Igh recombination (Zanotti et al., 2015). Immunoglobulin J chain (IgJ) is a small polypeptide that serves to regulate polymer formation and secretory activity of Immunoglobulin A (IgA) and Immunoglobulin M (IgM) (Brandtzaeg, 1983). IgE is produced primarily in response to parasitic infections and can also be involved in allergic reactions (Larsson et al., 2020). IGHE encodes a constant region of the IgE heavy chain (Wagner, 2009). Genes (BLNK, MYB, BTK, FCRL1, FCRL5, MSH2, PAX5, BCL11A) related to B cell activation were down-regulated in geriatric individuals. PAX5 is essential for many aspects of B cell development including immunoglobulin rearrangement, pre-B cell receptor signaling and maintaining cell identity in mature B cells (Holmes, Pridans & Nutt, 2008). We found that BLNK was a hub gene in our protein–protein interaction analysis. BLNK is a central linker protein connecting B cell receptor-associated kinases with multiple signaling pathways, which regulates the pre-BI to pre-BII transition and Ig light chain gene rearrangement, playing a non-redundant key function in B cell development (Lagresle-Peyrou et al., 2014). This suggests that BLNK may play a critical role in the progression of age-related immune changes in the red panda. These results show that the humoral immunity of red pandas increases then decreases with age. Taken together, we conclude that the adaptive immunity of red pandas increases then decreases with age.

### Gene expression changes associated with disease

The main cause of death in adult and geriatric red pandas was cardiovascular disease, and the proportion of death from cardiovascular disease in juveniles (31–365 days), adults (1–10 years) and geriatrics (>10 years) was 2.94%, 20.65% and 24.62%, respectively (Delaski, Ramsay & Gamble, 2015). Age is the biggest risk factor for cardiovascular disease, and the prevalence increases sharply with age. In geriatric individuals, we observed an up-regulation of PTX3, a prototypic long pentraxin, which is produced by somatic cells and innate immune cells after pro-inflammatory stimulation. In peripheral blood tissue, PTX3 was found to be significantly elevated in human patients with heart failure and acute myocardial infarction, contributing to the occurrence of cardiovascular events and cardiovascular risk factors (Inforzato et al., 2015; Liu et al., 2011; Peri et al., 2000). Therefore, PTX3 may be an effective target point for the prevention and treatment of cardiovascular disease in red pandas. The aging process and its related diseases often cause energy metabolism disorder and oxidative damage (Mattson, Maudsley & Martin, 2004). The most significant up-regulated gene, CP, between geriatric and adult red pandas, encodes a serum ferroxidase ceruloplasmin that is related to iron metabolism (Hellman & Gitlin, 2002). Clinical and epidemiological studies have shown that iron metabolism plays an important role in multiple aging-associated disorders, especially in cardiovascular disease (Roijers et al., 2011). Higher levels of serum ceruloplasmin, as an independent risk factor for cardiovascular disease, can be a marker for metabolic stresses associated with metabolic syndrome (Kim et al., 2002).

Vaccines appear as the most efficient and economical medical interventions against infectious diseases and have also been used to defend against pathogen infections in red pandas (Dumpa et al., 2019; Qin et al., 2007). However, some vaccines for red pandas have not produce consistent antibody titers, thus the protective effect did not meet expectations (Loeffler et al., 2007; Zhang et al., 2017) especially in geriatric individuals. One reason for this may be a decline in immune system function associated with aging and leads to a low vaccine response and vaccine longevity as well as reducing the preventive effectiveness of vaccination (Giefing-Kroll et al., 2015; van den Berg et al., 2018). Reduced adaptive immunity leads to reduced vaccine responses and vaccine longevity (Lord, 2013). Flagellin can trigger an innate immune response by interacting with a cell surface receptor TLR5 (Uematsu et al., 2006), subsequently stimulating T cell responses and initiating adaptive immunity (Applequist et al., 2005). In both murine models and clinical trials, incorporating flagellin into vaccines has been shown to enhance vaccination efficiency in the old (Leng et al., 2011; Taylor et al., 2011). Our study has indicated that TLR5 was significantly up-regulated in geriatric red pandas, suggesting the possibility of enhancing the immune response to vaccination by incorporating flagellin, via the TLR5 innate immune pathway to address the problem of decreased vaccine response. This research provides an orientation for us to develop effective vaccines for endangered animals such as red pandas in the future. This work provides an insight into gene expression changes associated with aging, and paves the way to develop effective disease prevention and treatment strategies for red pandas in the future.

## Conclussion

We have undertaken the first blood transcriptome analysis of aging red pandas. We found 2,219 DEGs between geriatric and adult individuals, and 1,690 DEGs between adults and juveniles. The gene expression and functional annotation results showed the innate immunity of red pandas significantly increased in geriatric individuals, but its change remains unclear in from juveniles to adults. We found the adaptive immunity of red pandas first increased and then decreased with aging. Our study is a first step to clarify the gene expression profiles in aging red pandas and provides an insight into aging gene expression changes. However, any innate immunity changes from juvenile to adulthood need further exploration. The treatment strategy of decreased vaccine response caused by age-related decline in immune system function, also need further research.

As an ideal surrogate tissue for diagnostics, the functional relevance of blood transcriptome studies as a method of measuring aging and immunity cannot be underestimated. However, considering the possible tissue and species specificity in gene function, and the small sample size in this study, the limitations of this study still exist.

##  Supplemental Information

10.7717/peerj.13743/supp-1 Supplemental Information 1 Basic information of samples

10.7717/peerj.13743/supp-2 Supplemental Information 2 The top 20 most significantly DEGs with annotation

10.7717/peerj.13743/supp-3 Supplemental Information 3 GO and KEGG enrichment of DEGs and other information

10.7717/peerj.13743/supp-4 Supplemental Information 4 Enrichment of DEGs and other information

## Additional Information and Declarations

### Data Availability

The following information was supplied regarding data availability: The data that support the findings of this study are available at the CNGB Sequence Archive (CNSA) (Guo et al., 2020) of China National GeneBank DataBase (CNGBdb) (Chen et al., 2020a): CNP0002175.
