# Supplementary material for: Blood transcriptome analysis revealing aging gene expression profiles in red panda
Source: PeerJ. 2022 Jul 22;10:e13743. doi: 10.7717/peerj.13743 (PMC9310792; doi:10.7717/peerj.13743)
Supplement: Supplemental Information 3 [file peerj-10-13743-s003.pdf]

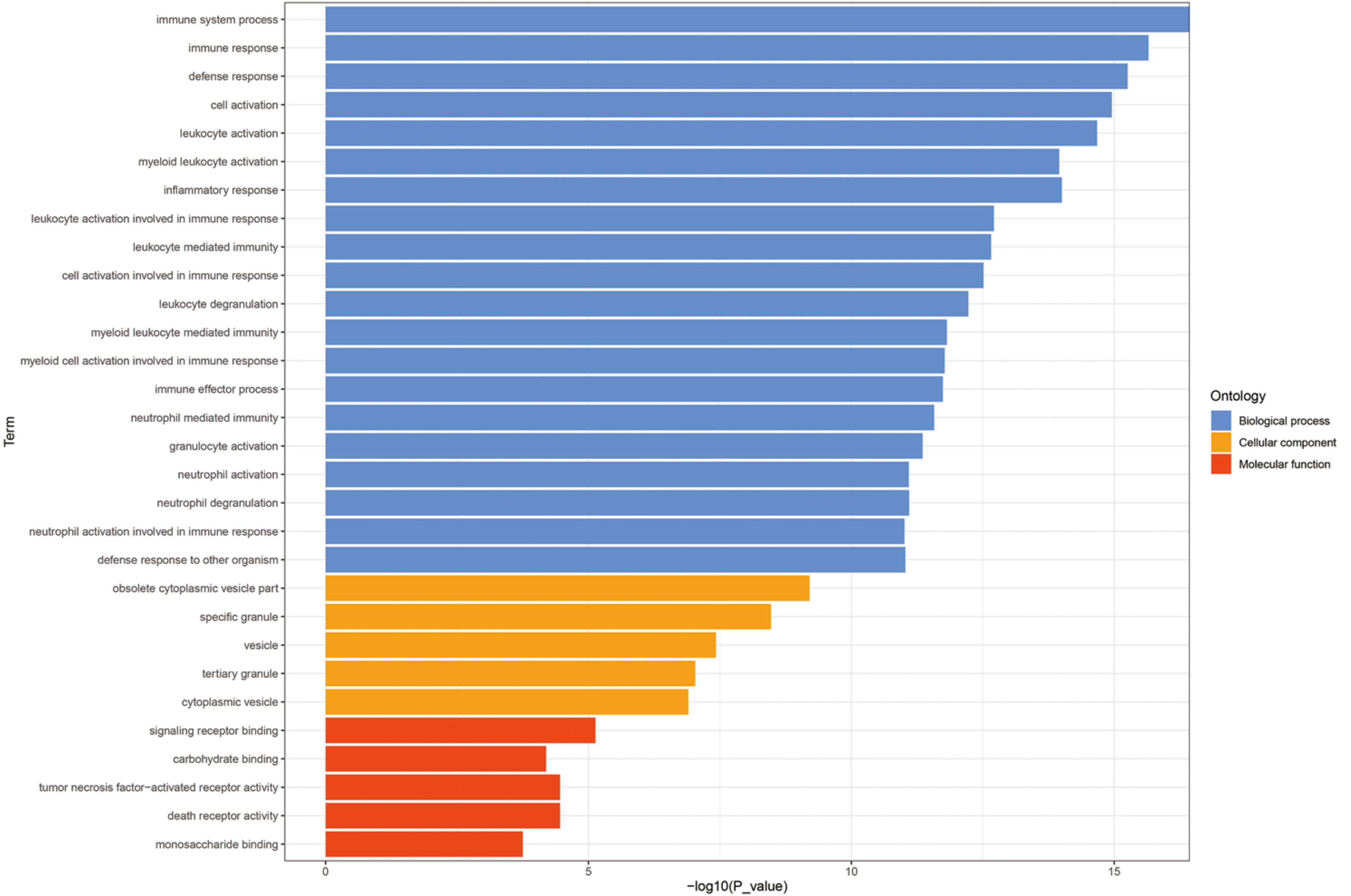

GO enrichment analysis of up-regulated DEGs between geriatric and adults.

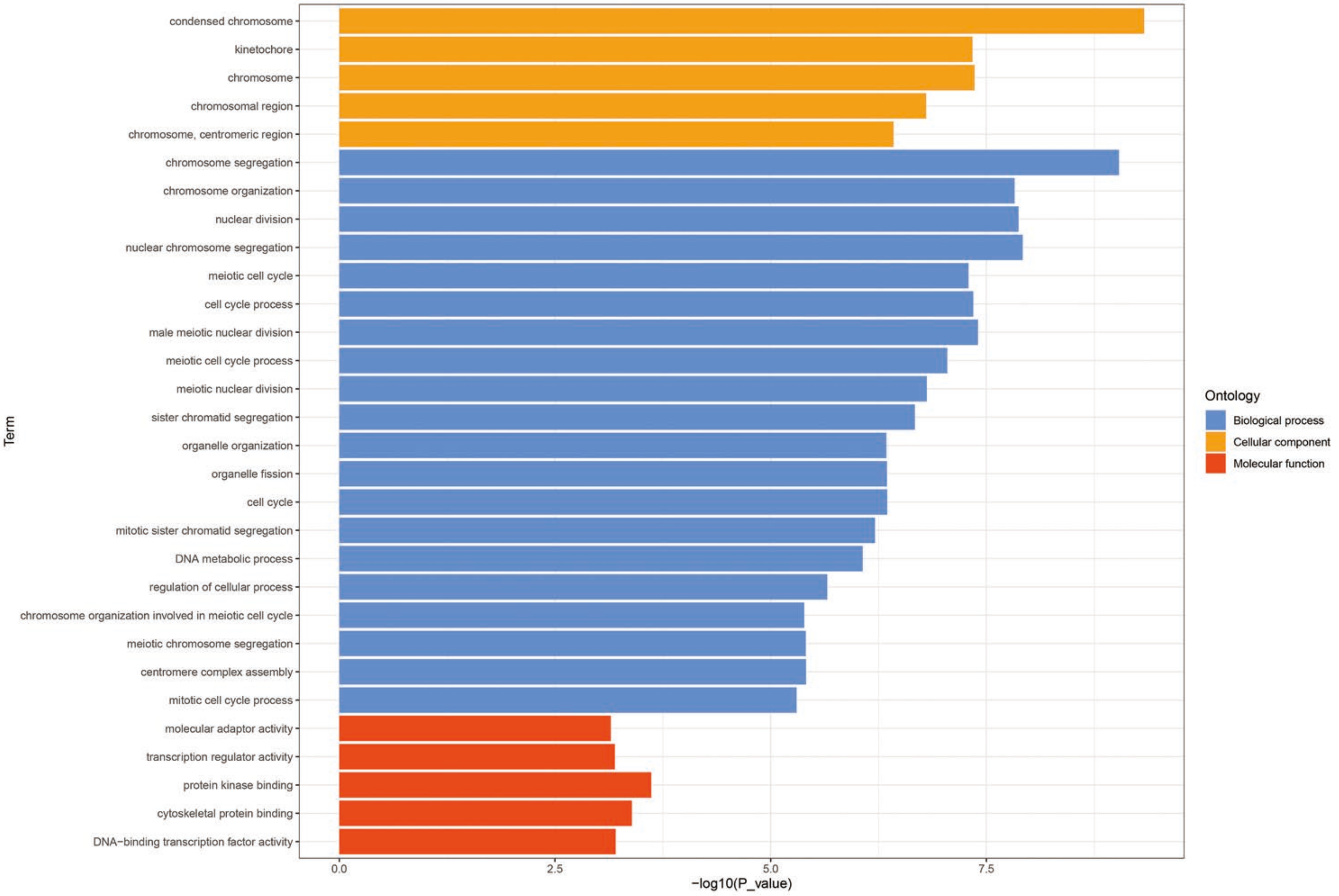

GO enrichment analysis of down-regulated DEGs between geriatric and adults.

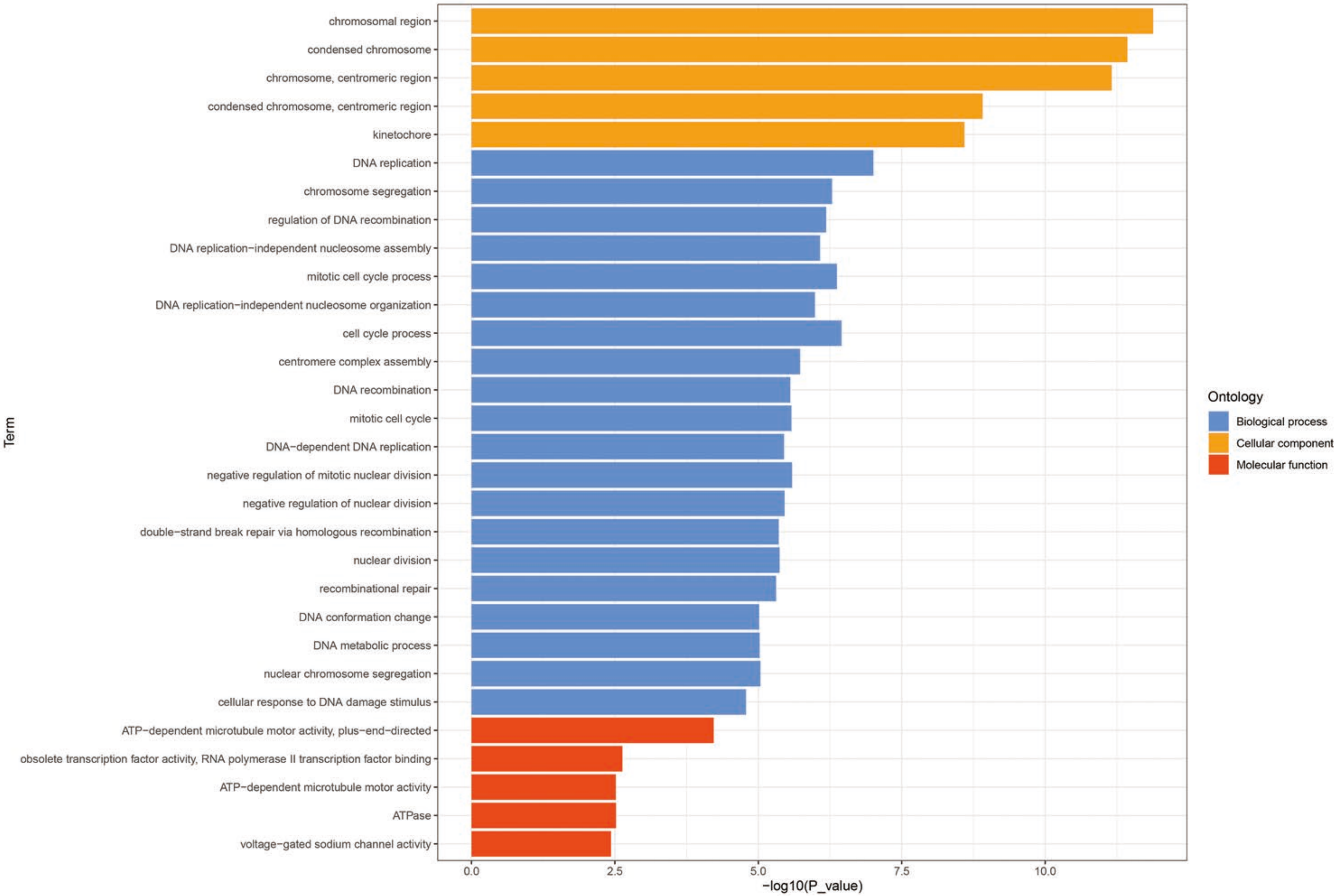

GO enrichment analysis of up-regulated DEGs between adults and juveniles.

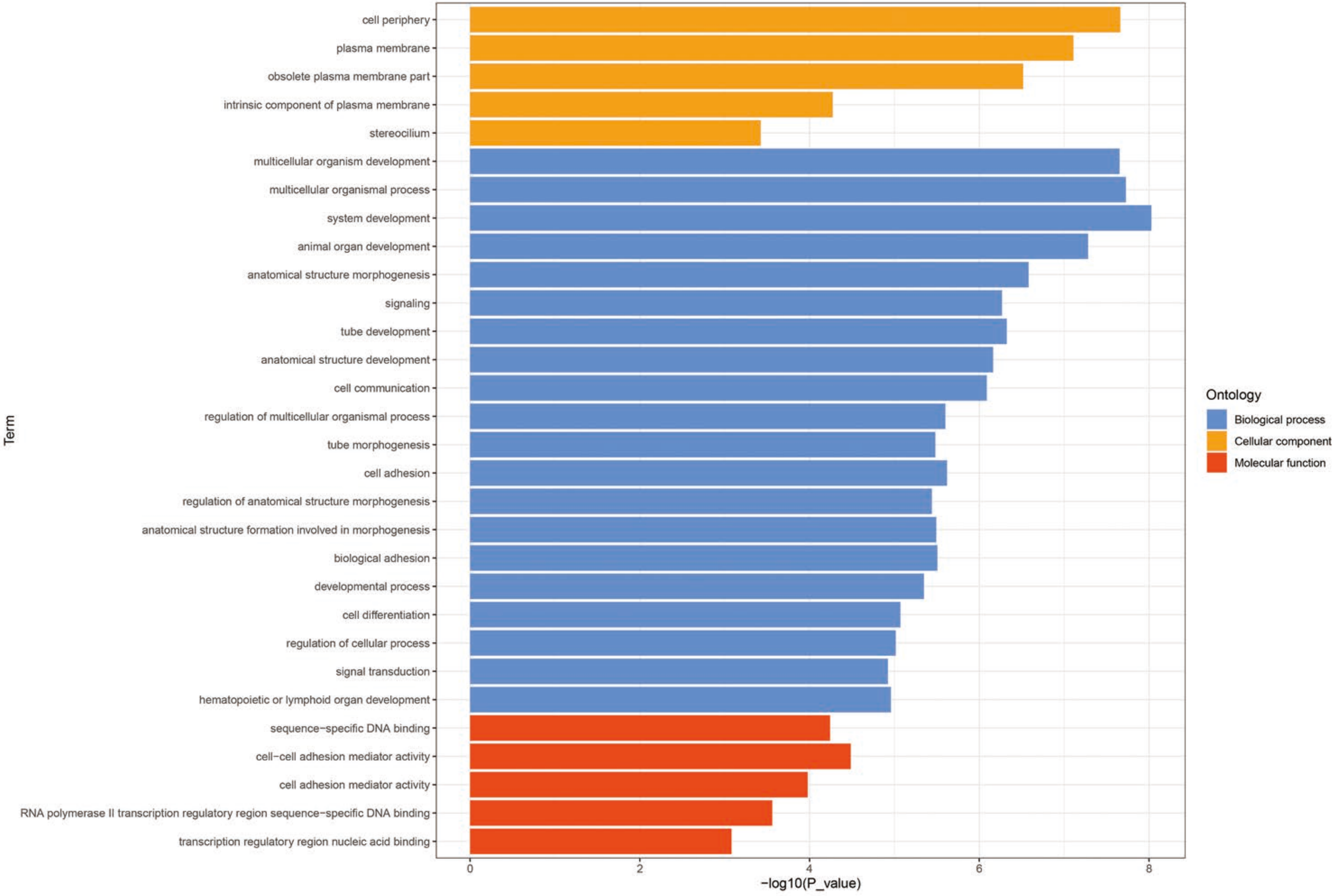

GO enrichment analysis of down-regulated DEGs between adults and juveniles.

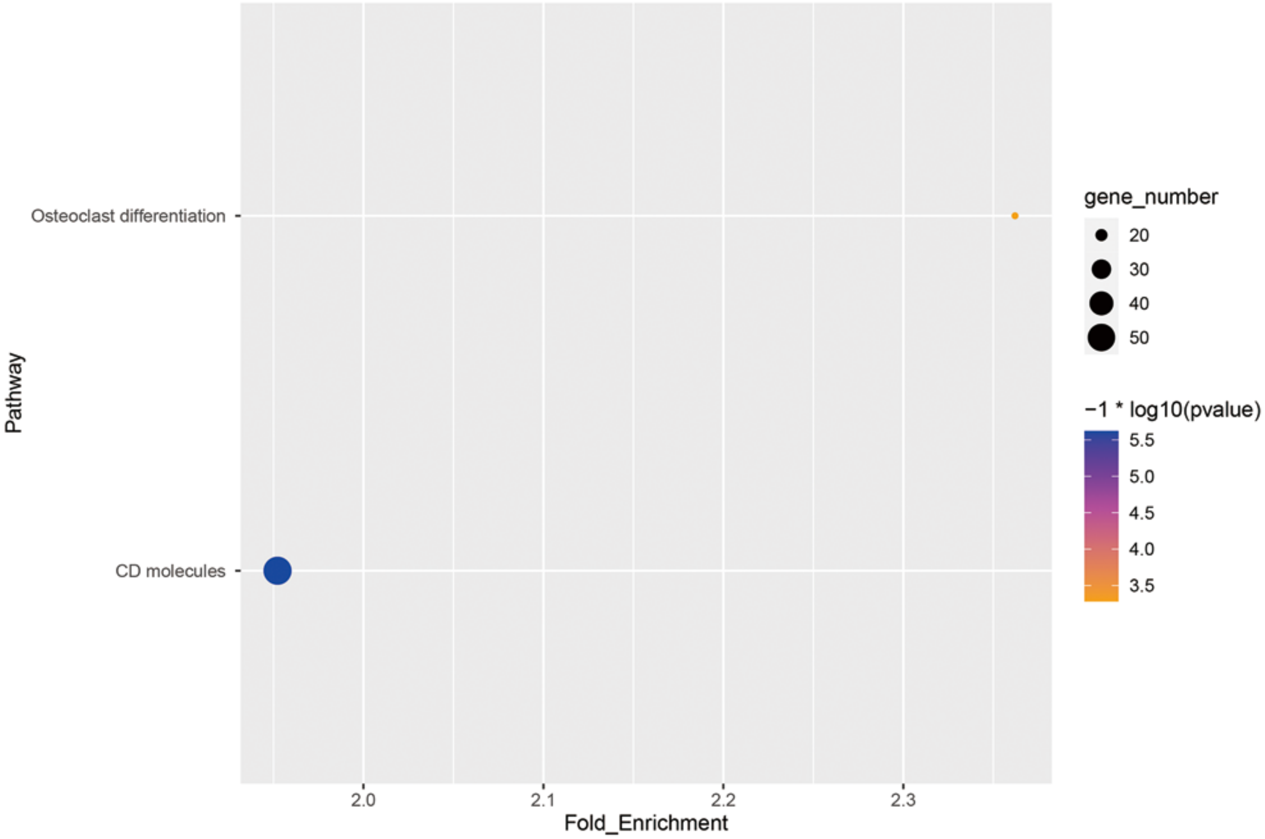

KEGG enrichment analysis of up-regulated DEGs between geriatric and adults.

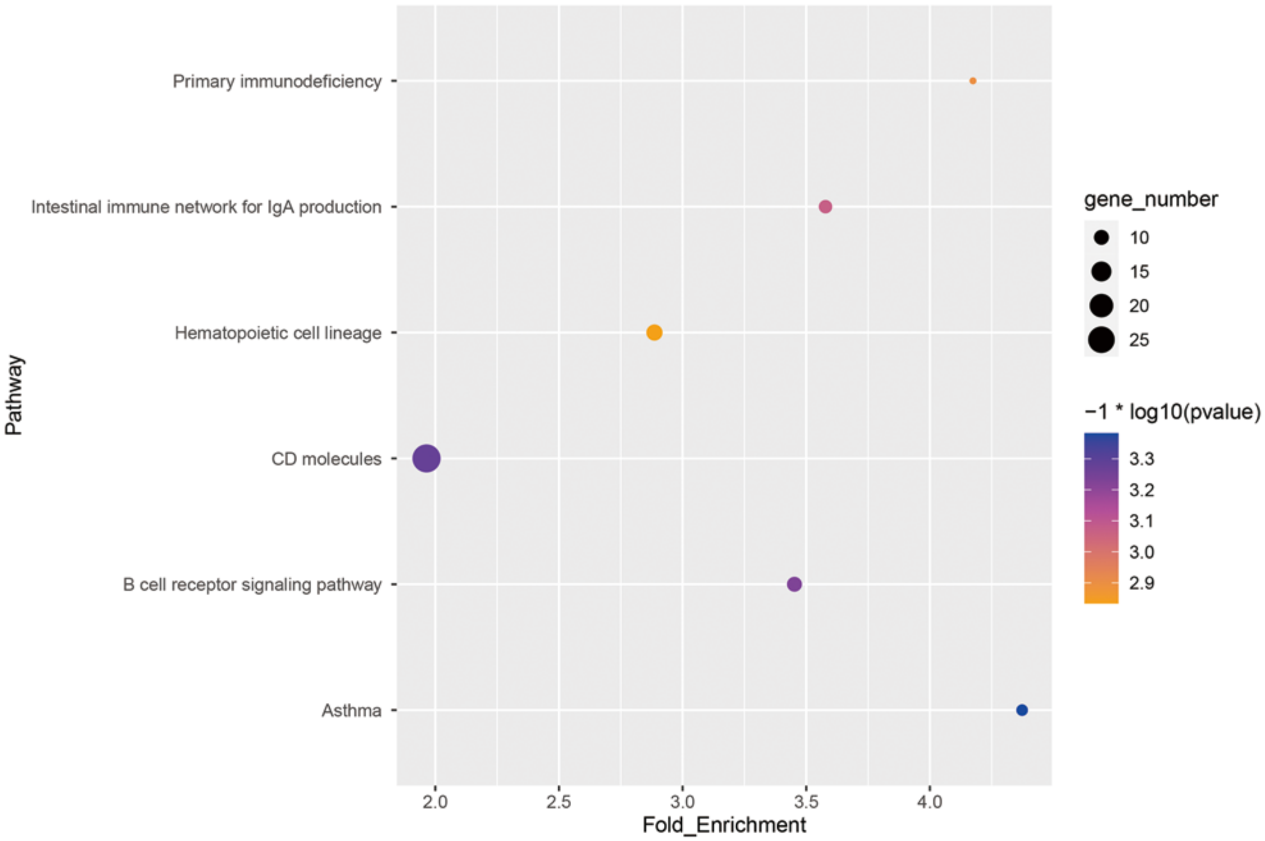

KEGG enrichment analysis of down-regulated DEGs between geriatric and adults.

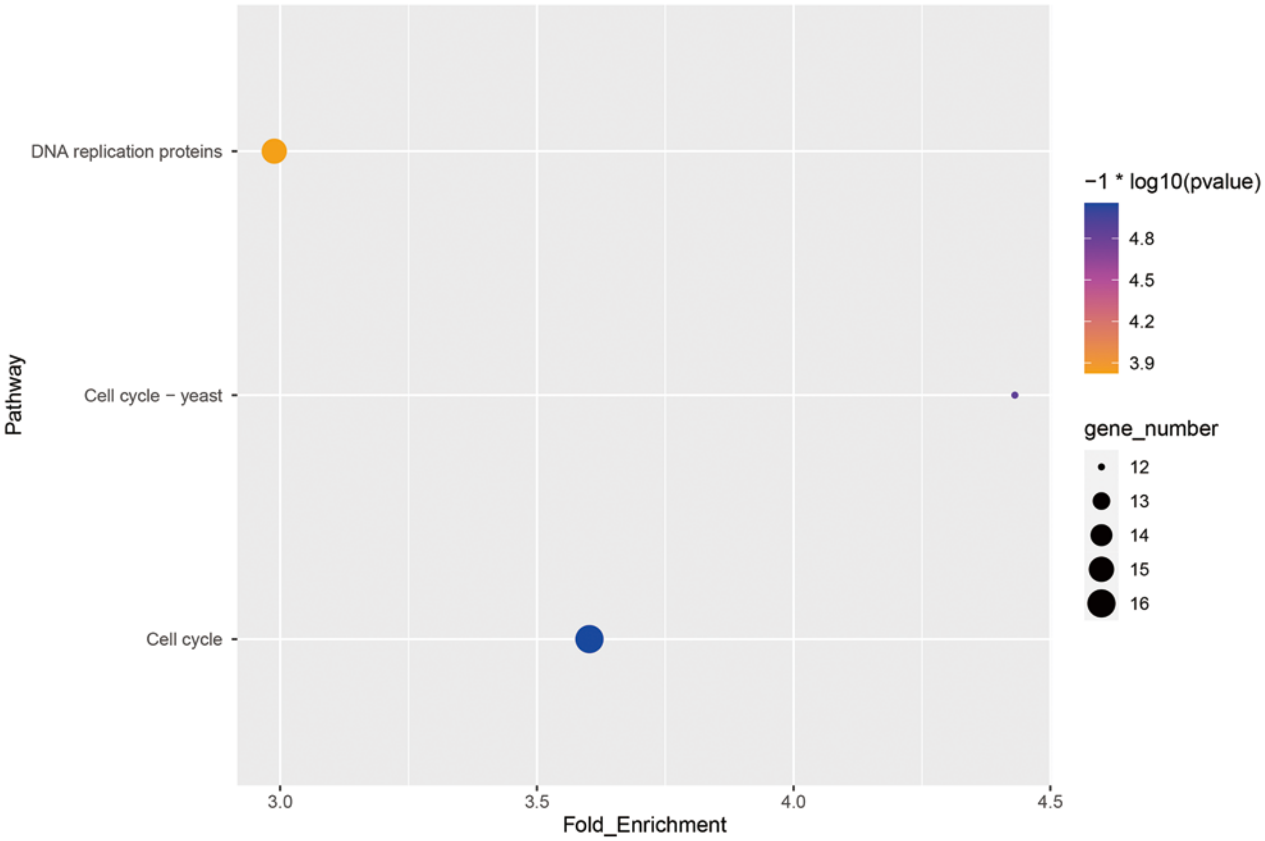

KEGG enrichment analysis of up-regulated DEGs between adults and juveniles.

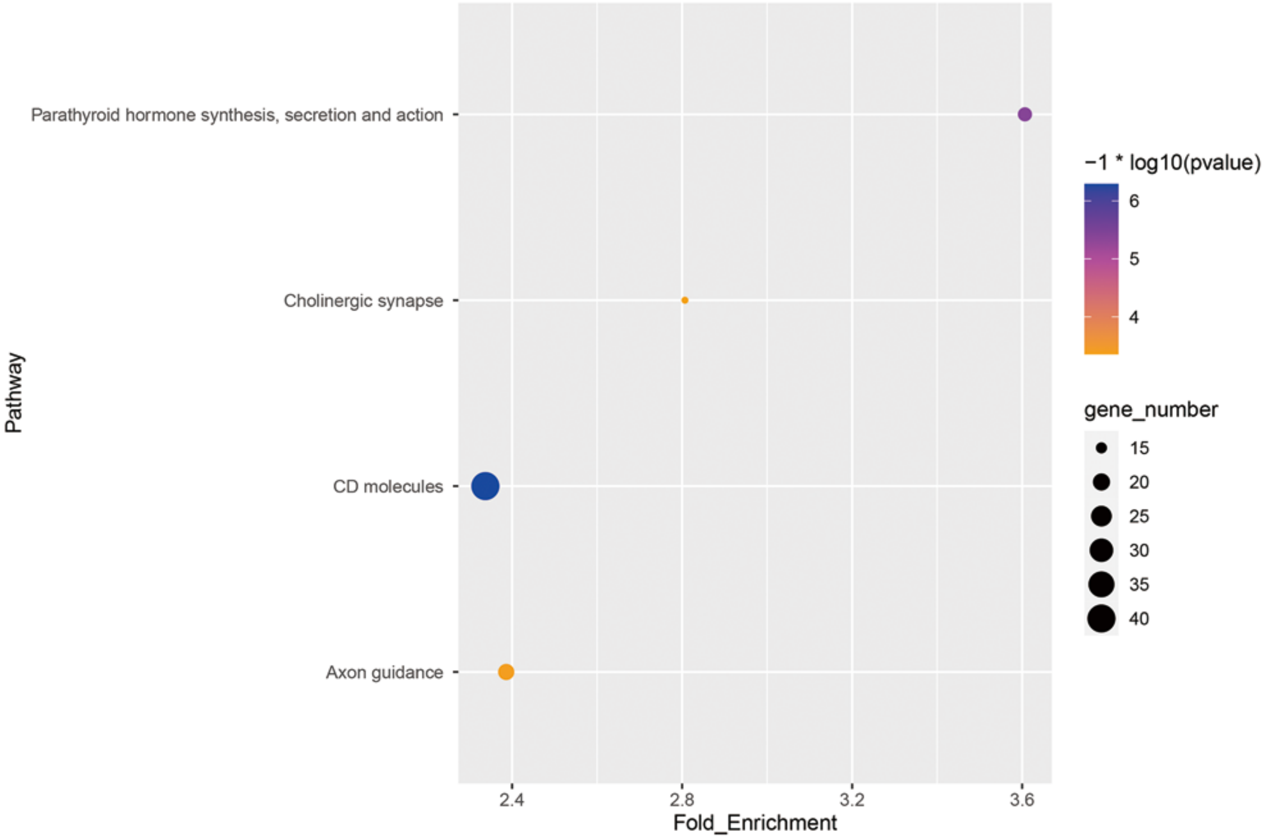

KEGG enrichment analysis of down-regulated DEGs between adults and juveniles.

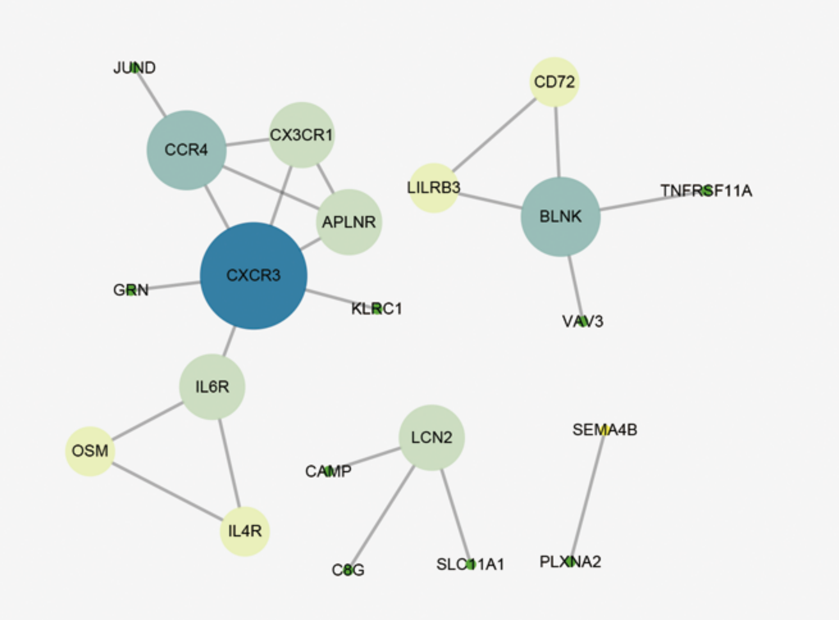

protein-protein interaction analysis of 33 immune-related DEGs that were common in the two comparisons.

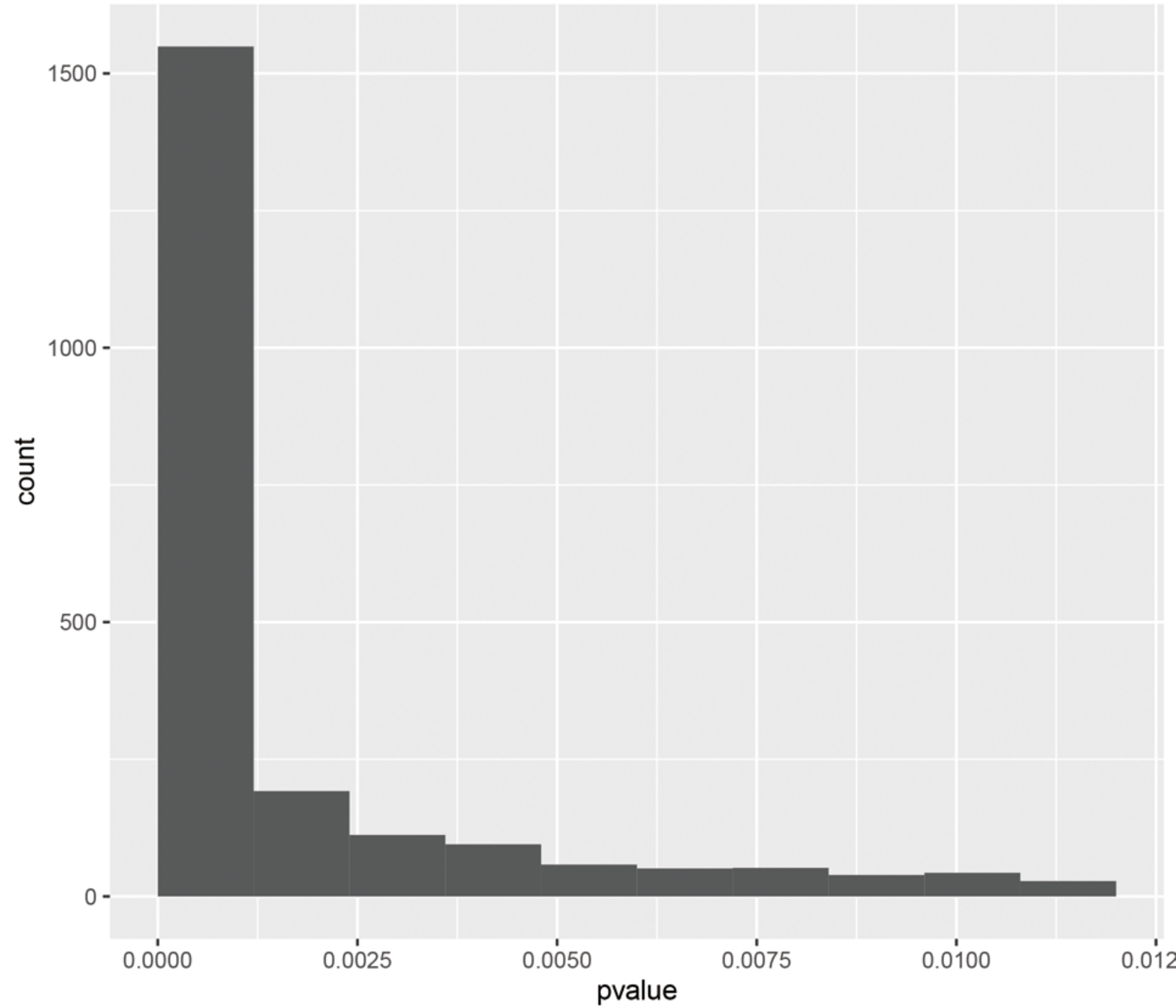

P-values frequency distribution of DEGs between geriatric and adults.

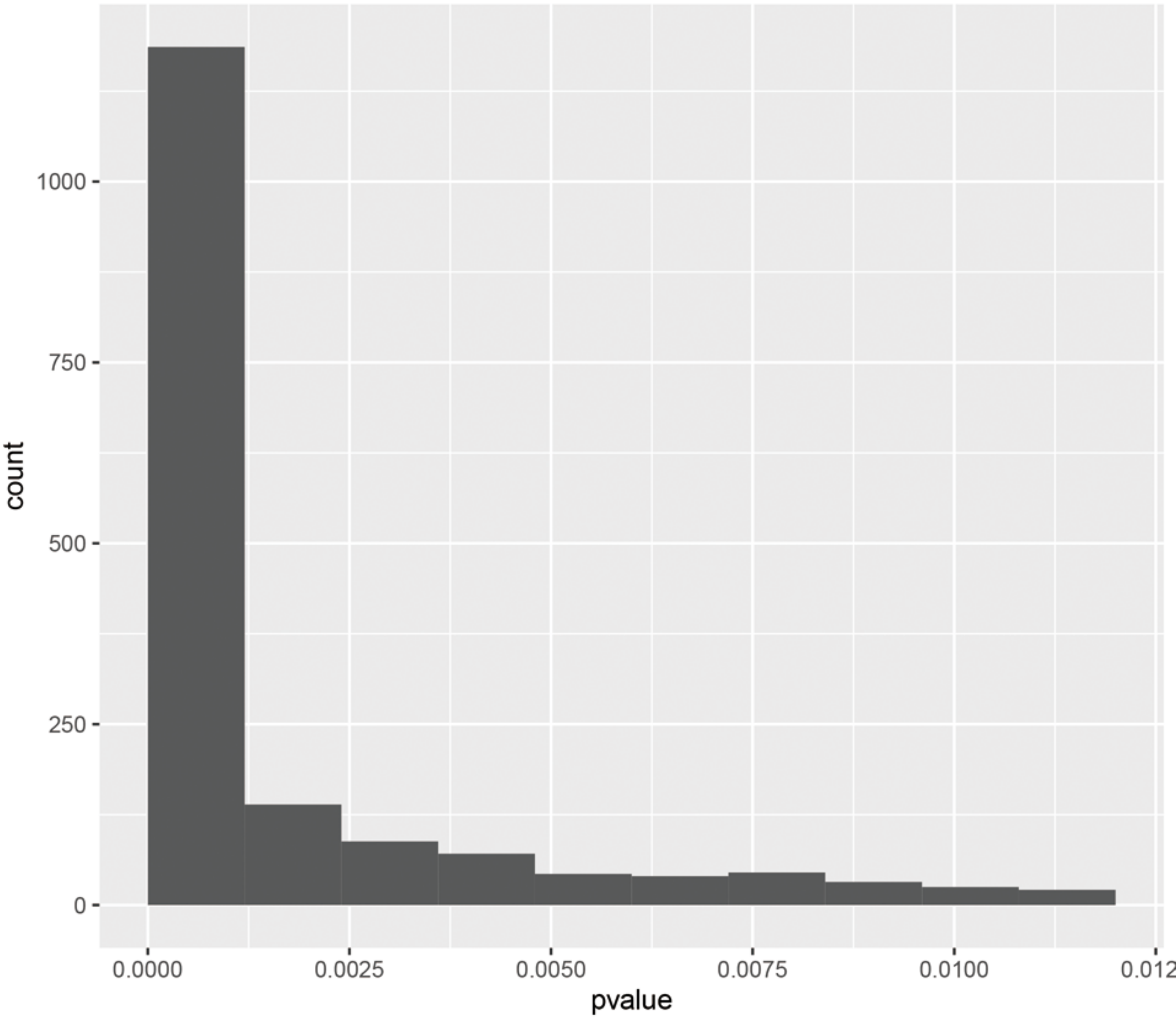

P-values frequency distribution of DEGs between adults and juveniles.
